# Editorial: Animal-borne viral disease: pathogenesis, innate immunity, acquired immunity, and novel vaccine development

**DOI:** 10.3389/fimmu.2025.1713564

**Published:** 2025-10-29

**Authors:** Lei Tan, Fulong Nan, Shen Yang

**Affiliations:** ^1^ Shanghai Veterinary Research Institute, Chinese Academy of Agricultural Sciences, Shanghai, China; ^2^ Department of Veterinary Medicine, Qingdao Agricultural University, Qingdao, China; ^3^ Department of Medicine, University of California, San Diego, La Jolla, CA, United States

**Keywords:** animal-borne viral diseases, pathogenesis, innate and adaptive immunity, vaccine development, diagnostic innovations

Animal-borne viruses present a significant threat to global animal health and the livestock industry, resulting in annual economic losses amounting to tens of billions of dollars. This Research Topic presents a collection of studies focused on a range of pathogens—from avian influenza and porcine viruses to emerging zoonotic agents—addressing key themes such as pathogenesis, innate and adaptive immunity, and novel vaccine development. Together, these articles elucidate the mechanisms by which host–pathogen interactions shape disease outcomes and demonstrate how advanced immunological tools can be leveraged to counter animal-borne viral threats. The articles presented here highlight novel vaccine platforms, host immune responses and pathogenesis, advances in diagnostics, and broad perspectives derived from comprehensive reviews.

## Innovative vaccine platforms and immunization strategies

Vaccination continues to serve as the cornerstone of infectious disease control, and multiple studies in this Research Topic highlight advances in vaccine technology. For instance, Tang et al. developed a feline herpesvirus type 1 (FHV-1) vector delivering antigens from feline calicivirus (FCV) and feline parvovirus (FPV). Using CRISPR/Cas9-mediated editing, the team generated a recombinant FHV-1 strain co-expressing FCV VP1 and FPV VP2, which induced robust neutralizing antibodies and conferred complete protection in cats against challenge with both FHV-1 and FPV. This proof-of-concept study supports FHV-1 as a promising multivalent vaccine platform for important feline pathogens.

Similarly, novel nucleic acid and nanoparticle-based vaccines show substantial potential for swine diseases. Liu et al. developed a circular mRNA-LNP vaccine encoding the classical swine fever virus (CSFV) E2 glycoprotein fused to a self-assembling mi3 nanoparticle induced markedly stronger immune responses than a conventional subunit vaccine. In mice, the E2-TMD-mi3 mRNA-LNP formulation elicited significantly higher antibody blocking rates (∼80%) and a fivefold increase in IFN-γ–producing T-cell responses compared with controls. In immunized pigs, antibody levels remained above the protective threshold for months after vaccination. These findings underscore how mRNA platforms enhanced by engineered nanoparticle scaffolds and modern lipid formulations can overcome the limited immunogenicity of protein-based vaccines.

Gao et al. showed that nanoparticle-based delivery systems also improve T cell–mediated protection against influenza. Liu et al. encapsulated plasmid DNA encoding influenza hemagglutinin (HA) into silica–calcium phosphate nanoparticles (approximately 226 nm in diameter). In mice, these nanoparticles stimulated sustained CD4^+^ and CD8^+^ T-cell responses detectable up to 12 weeks after vaccination, whereas conventional vaccines typically induce only short-lived cellular immunity. Following a lethal challenge with a heterologous H3N2 strain, all mice immunized with nanoparticles carrying HA from one influenza strain survived, and a construct with a different HA still provided significant protection, with 66% survival. These results indicate that nanoparticle-delivered DNA vaccines offer a promising strategy for achieving broad and durable influenza immunity.

In addition to genetic vaccine platforms, several studies explored subunit and inactivated vaccines with advanced formulations. Yang et al. developed a virus-like particle (VLP) vaccine for feline calicivirus (FCV) using the VP1 capsid gene from a broadly neutralizing FCV strain. In challenge studies, cats immunized with these VLPs exhibited complete protection, showing no clinical signs and demonstrating markedly reduced viral shedding and viremia. This VLP strategy overcomes safety limitations associated with live or inactivated vaccines by inducing potent neutralizing immunity through non-replicating particles. In another vectored approach, Findlay-Wilson et al. evaluated soluble glycoproteins from Nipah virus (NiV) and Hendra virus (HeV) in a hamster model. Immunization with either glycoprotein—delivered with adjuvant—elicited cross-reactive humoral immunity, and all immunized hamsters survived a lethal NiV challenge. This finding indicates that a single henipavirus antigen can confer cross-protection against related viruses, supporting the potential for a pan-henipavirus vaccine suitable for outbreak response.

Beyond viral targets, advanced inactivation methods are also broadening vaccine possibilities for bacterial diseases that involve viral-like pathogenesis. In one study, Belay et al. inactivated Pasteurella multocida, the causative agent of fowl cholera, using gamma irradiation. When formulated with Th1-biasing adjuvants, the irradiated vaccine provoked high levels of serum IgG and IgA, along with potent T-cell cytokine responses—including a remarkable >1000-fold upregulation of IFN-γ—in chickens. All vaccinated birds were fully protected against a lethal homologous challenge. This study underscores how alternative inactivation methods, such as gamma irradiation compared to traditional formalin treatment, can stimulate comprehensive immunity encompassing mucosal, humoral, and cellular arms.

Collectively, the articles in this Research Topic demonstrate cutting-edge advances in vaccine development against animal-borne viruses, spanning viral vectors, nucleic acid platforms, nanoparticles, and virus-like particles. They target a wide range of pathogens—feline, porcine, avian, and zoonotic—and highlight a clear trend toward gene-based platforms combined with rationally designed antigens and adjuvants. The innovations presented provide a strong foundation for the next generation of animal immunoprophylaxis.

## Host immune responses and disease pathogenesis

Understanding how animal hosts respond to viral infection—including both protective immunity and immunopathology—is essential for developing effective immunotherapies and informing the breeding of disease-resistant livestock. Several articles in this Research Topic dissect immune dynamics following viral challenge. In one study, Park et al. compared two highly pathogenic Korean strains of porcine reproductive and respiratory syndrome virus type 2 (PRRSV-2) with a vaccine-like strain in piglets. The most virulent isolate (PJ10) induced high mortality and severe pulmonary and cerebral lesions, accompanied by near-complete depletion of alveolar macrophages and substantial infiltration of immune cells. Notably, surviving animals exhibited increased expression of immune checkpoint molecules—including PD-1, PD-L1, CTLA-4, IDO1, and LAG3—on bronchoalveolar lymphocytes. This upregulation coincided with impaired T-cell activation, suggesting that PRRSV may exploit checkpoint pathways to suppress T-cell function as an immune evasion mechanism. These observations offer important insights into PRRSV pathogenesis, illustrating how inter-strain genetic differences shape immunopathological outcomes and supporting the need for control strategies that can counteract virus-induced immunosuppression.

In the context of African swine fever virus (ASFV) in swine, Friedrichs et al. evaluated the protective role of passive maternal immunity in piglets. Although piglets born to ASFV-recovered sows possessed maternal antibodies, they succumbed rapidly to a highly virulent ASFV challenge. Neither colostral transfer nor artificial IgG infusion prolonged survival beyond nine days post-infection, with all piglets reaching humane endpoints. This sobering outcome indicates that antibodies alone are insufficient to protect neonates against ASFV. Protective immunity likely requires robust cellular responses, underscoring the limitation of humoral transfer and informing future efforts to develop effective ASFV vaccines.

Yang et al. identified a novel microRNA-mediated pathway that connects viral sensing to oxidative stress management. Yu et al. report that miR-1985, induced by poly(I:C), suppresses a scallop MNK1 homolog, thereby increasing antioxidant enzyme activity and protecting against PAMP-induced stress. This miR-1985/PyMNK1/SOD/catalase pathway represents an important immunoregulatory mechanism, highlighting an evolutionarily conserved strategy for balancing antiviral defense with damage control in invertebrates.

In humans and other vertebrates, viral infections can trigger maladaptive immune responses that contribute significantly to tissue damage and pathology. Moragas et al. analyzed lung tissue from children with fatal dengue virus infection, observing extensive pulmonary injury—including hemorrhage, vascular congestion, and edema—alongside dense infiltration of mononuclear cells. Immunohistochemical staining confirmed active dengue virus replication within the lungs, accompanied by elevated levels of pro-inflammatory cytokines. These results support a model of lethal immunopathology in pediatric dengue, in which vascular leakage and dysregulated inflammation—rather than viral load alone—are the primary drivers of mortality.

Collectively illuminating the interplay between host immunity and viral pathogenesis, these studies significantly advance the understanding of innate and adaptive defense mechanisms and their subversion by pathogens. Research highlighted in this section spans innate sensing, adaptive responses, and immune evasion, clarifying regulatory pathways from miRNA signaling to checkpoint expression. These insights provide a framework for developing targeted interventions that enhance host resistance and guide the design of effective vaccines.

## Diagnostic and serological innovations

Accurate diagnostics are fundamental to effective surveillance and control of animal viruses. Addressing this need, two original studies in this Research Topic present rapid, high-fidelity serological assays. Wang et al. developed a pseudovirus-based neutralization test for transmissible gastroenteritis virus (TGEV), a swine coronavirus. By expressing the TGEV spike protein on a vesicular stomatitis virus (VSV) pseudotype, the team created a safe and reliable assay that demonstrated 100% sensitivity and 96.6% specificity relative to the live-virus neutralization test. The assay showed no cross-reactivity with other common porcine viruses and accurately tracked vaccine-induced antibody titers. This scalable system overcomes key limitations of conventional neutralization tests—such as lengthy viral culture and biosafety constraints—and is positioned to support large-scale monitoring of TGEV immunity.

In the bovine health sector, Qi et al. developed a novel competitive ELISA (cELISA) for detecting antibodies against bovine viral diarrhea virus (BVDV). The team generated monoclonal antibodies targeting the viral E2 glycoprotein and identified one monoclonal antibody (3E6) that recognizes a conserved epitope within domain B of E2, common to both BVDV-1 and BVDV-2 genotypes. The resulting cELISA exhibited high diagnostic performance, with 99.26% sensitivity and 98.99% specificity, and showed no cross-reactivity with other common livestock viruses. Notably, the assay demonstrated strong concordance with virus neutralization tests when evaluated on hundreds of field samples. This pan-genotypic serological tool provides a reliable and scalable method to enhance BVDV surveillance and facilitate vaccine efficacy evaluation.

The developments outlined above highlight how contemporary molecular techniques are yielding diagnostics for animal pathogens. These new serological tools provide critical capabilities for mass screening and epidemiological vigilance, creating synergies with vaccine programs and collectively advancing the strategic goal of implementing innovative solutions for disease control.

## Insights from emerging pathogen studies

This Research Topic also features comprehensive overviews of several high-consequence zoonotic viruses. Chen et al. provided a review on Severe Fever with Thrombocytopenia Syndrome virus (SFTSV) underscores that although the disease carries a high case-fatality rate (up to 30%) in East Asia, no existing animal model fully recapitulates its manifestations in humans. Although several candidate vaccines—including mRNA, subunit, and viral vector platforms—have shown promise in preclinical studies, their development must still overcome challenges such as viral genetic diversity and immune evasion mechanisms. As emphasized by the authors, advancing a broadly effective SFTSV vaccine will require more physiologically relevant animal models and a deeper integration of immunologic and virologic insights. Complementing this, a review by Berihulay et al. on Newcastle disease virus (NDV) consolidates decades of research to provide a scientific foundation for breeding genetically resistant poultry and for directing the next generation of NDV vaccines.

In summary, this topic of articles highlights both the complexity and urgency of combating animal-borne viral diseases. By illuminating immune mechanisms—from initial pathogen recognition to adaptive and regulatory responses—and pioneering novel vaccine and diagnostic technologies, these contributions collectively outline a pathway toward improved prevention and control of livestock and zoonotic infections. The advances compiled here, unified by a focus on host–pathogen interactions, help set the stage for developing more effective vaccines and antiviral therapies. The diversity of findings—ranging from feline herpesvirus–vectored vaccines and swine mRNA platforms to redox-mediated antiviral signaling in mollusks and immunopathology in human dengue—reflects the editorial objective of integrating basic mechanistic research with translational innovation. Such synergy between fundamental immunology and cutting-edge prophylactic tools will be crucial for protecting animal health and strengthening public health preparedness against emerging viral threats.

